# Supplementary material for: Opposite Nuclear Dynamics of Two FRH-Dominated Frequency Proteins Orchestrate Non-Rhythmic Conidiation in Beauveria bassiana
Source: Cells. 2020 Mar 5;9(3):626. doi: 10.3390/cells9030626 (PMC7140403; doi:10.3390/cells9030626)
Supplement: Supplementary file 1 [file cells-09-00626-s001.pdf]

Supplementary Materials

Table S1. Paired primers used for manipulation of target genes and identification of their mutants in *B. bassiana*.

Primers	Sequences (5′–3′) *	Purpose
Frq1-F/R	AGCTTTGTTTAAACATGCCCAAGACGCAGATAAACACT / AAAAAGACTAGTCTCCTCATCGTCTTCACTGCTGCT	Cloning *frq1* cDNA (2968 bp) for fusion to *GFP*
Frq2-F/R	AGCTTTGTTTAAACATGACCAGCGGCGCCTCACT / AAAAAGGAATTCCCACTCTGCATTATTCAGACATG	Cloning *frq2* cDNA (1753 bp) for fusion to *GFP*
Frh-F/R	AGCTTTGTTTAAACATGGACGAAATGTTTGATGTGTT / AAAAAGACTAGTCTACAGGTACAGACTCTGCGCA	Cloning *frh* cDNA (3297 bp) for fusion to *GFP*
Frq1up-F/R	AAACCGGAATTCGAAATCCTGTTCCAAGCCAAAT / AAACGCGGATCCAAAGCAAGCAAGGGTCCAAG	Cloning *frq1* 5′ fragment (1360 bp) for recombination /deletion
Frq1dn-F/R	AAAAGCTCTAGACAACCACGACATCAAGAAACG / AACGCCGTTAACGAGTCCTGCCGAACAAAGAG	Cloning *frq1* 3′ fragment (1406 bp) for recombination /deletion
Frq1fl-F/R	GGGGACAAGTTTGTACAAAAAAGCAGGCTTGTTCGCCGTTGTCATCTATTT / GGGG ACCA CTTTGTAC AAGAAAGCTGGGTCTCCCGTCTTTCCTTGATTCTT	Cloning full-length *frq1* (7099 bp) for complementation
Frq2up-F/R	AAACCCAAGCTTAATGTTGTAGCCTGGTGGT / AAAAACGAGCTCCATAAGGTGCTGATGACGC	Cloning *frq2* 5′ fragment (1416 bp) for recombination /deletion
Frq2dn-F/R	AAAAGCTCTAGAATCGCTTTGCCGAACTGCT / AACGCCGTTAACCACCCGACTACTCGCCCTC	Cloning *frq2* 3′ fragment (1488 bp) for recombination /deletion
Frq2fl-F/R	GGGGACAAGTTTGTACAAAAAAGCAGGCTTGGGAATGGGACCTGAAACAC / GGGG ACCACTTTGTA CAAGAAAGCTGGGTAAGCAGCGGCAACAAGGACTA	Cloning full-length *frq2* (5613 bp) for complementation
Frhup-F/R	AAACCGGAATTCGGTCGGATTTGTAGGTTTGG / AAACGCGGATCCCTGATTTCGCTTGAGGCACT	Cloning *frh* 5′ fragment (1490 bp) for recombination /deletion
Frhdn-F/R	AAAAGGACTAGTGGAGGTTGAGGACAAGAAAGG / AACGCCGTTAACGCGGGTCTAGGATAGGCAGT	Cloning *frh* 3′ fragment (1384 bp) for recombination / deletion
Frhfl-F/R	GGGGACAAGTTTGTACAAAAAAGCAGGCTTGAAGGGAAGGTTGGTGTAGG / GGGGACCACTTTG TACAAGAAAGCTGGGTGGGCTTTGATAGGCTGTTGC	Cloning full-length *frh* (4789 bp) for complementation
pFrq1-F/R	TCATTTGCTTGCCGTCATCG / CTGCTGCTTTGCGTTTCTTG	PCR detecting *frq1*
pFrq2- F/R	GTGATAGCGGTATTGTTCC / TGCCCAGATACTTCTCAAC	PCR detecting *frq2*
pFrh-F/R	ACACCACAGAAGACACCGAAAT / TCAAGATAGGCAACAAACCAGA	PCR detecting *frh*
sbFrq1-F/R	ACAGCAACAAGCCAACGC / CGACCAACAGGCAGGAGA	Southern probe of *frq1*
sbFrq2-F/R	AGAACTGTGACCAGGGAGC / GGGCGTCGTTGTAGTAAAT	Southern probe of *frq2*
sbFrh-F/R	TAGGCAATCTTCCCGCATCA / CCCATTTCGGTGTCTTCTGTG	Southern probe of *frh*

* Underlined regions denote the introduced cleavage sites of restriction enzymes for fusing the *frq1* (*Pme*I/*Spe*I), *frq2* (*Pme*I/*Eco*RI) or *frh* (*Pme*I/*Spe*I) cDNA to *GFP*, inserting the 5′ and 3′ fragments of *frq1* (*Eco*RI/*Bam*HI and *Xba*I/*Hpa*I), *frq2* (*Hin*dIII/*Sac*I and *Xba*I/*Hpa*I) or *frh* (*Eco*RI/*Bam*HI and *Spe*I/*Hpa*I) into Δ*ura3* for targeted gene deletion through homogeneous recombination, and exchanging for the gateway fragments for targeted gene complementation, respectively.

Table S2. Paired primers used for transcriptional profiling of FRQ- and FRH-related genes in *B. bassiana*.

Tag locus*	Gene	Annotation	Sequences (5′–3′) of paired primers used in qPCR
BBA_04942	*fluG*	Transcription factor FluG	CCTCCCTAGTTTGGTCGCTTTCTC / CGCTGTCGGTAATCTGCTCCTC
BBA_07544	*brlA*	Key developmental activator BrlA	GACCAGTTCAACAGACAAG / CAGTAATCTTCGTGCTTCTC
BBA_00300	*abaA*	Developmental activator AbaA	GCAAGTCTCCAGCCATAT / CTCCTCTTCGTCATAGTAGTC
BBA_06126	*wetA*	Developmental activator WetA	CGCAGACGAATTTGACTT / GCTGGTGGTTGAATACAT
BBA_01528	*frq1*	Frequency clock protein Frq1	AACTCAATGGCATCAGAT / TATCCTTGTCCGTCATAG
BBA_08957	*frq2*	Frequency clock protein Frq2	CTCAGCATATACCTCCAT / CCAGATACTTCTCAACCT
BBA_02876	*vvd*	Blue-light receptor VIVID	ATTGCCAACTACAAGAAGA / GGAAGCCAACAGAGTATT
BBA_10271	*wc-1*	White Collar 1 protein WC-1	GCTATTTCATTGGCTTTCA / ATATCGCTGTGCTTGTAA
BBA_01403	*wc-2*	White Collar 2 protein WC-2	CATTACTACTGGCAATAC / ACATATTCCTCAGATACTT
BBA_02816	*phy*	Red/far-red light receptor PHY	TTATCGTCCGCTATGTTA / AGGAGATTGTCTTGTTGA
BBA_04860	*γ-actin*	γ-actin used as internal standard	TCTCTACGGCAACATTGTC / GCTGGAAGGTGGATAGG

* Gene accession codes in the *B. bassiana* genome under the NCBI accession NZ_ADAH00000000.

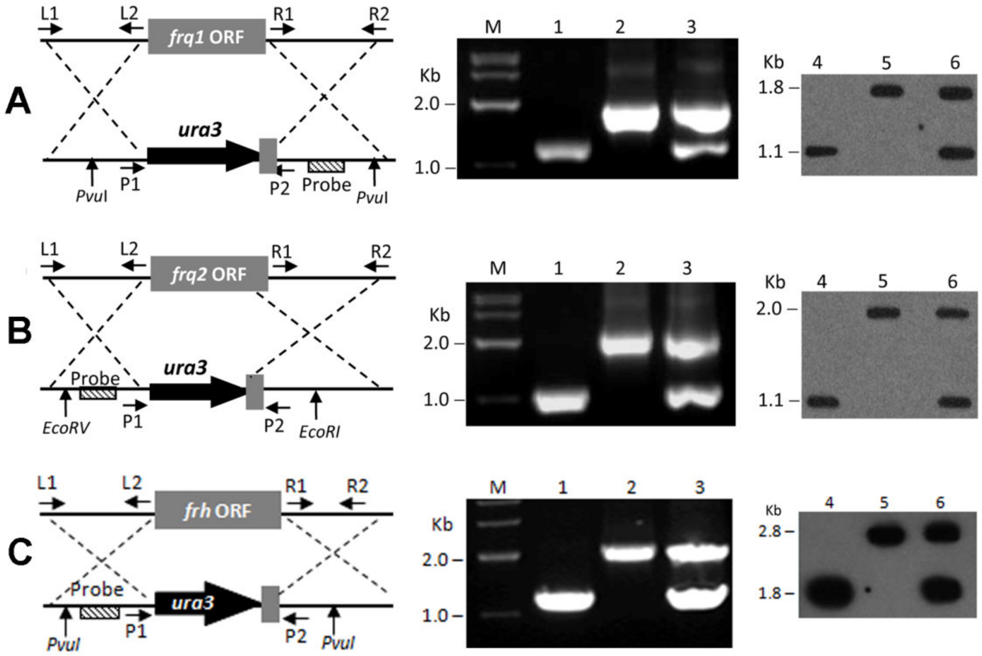

Figure S1. Generation and identification of *frq1*, *frq2* and *frh* mutants in *B. bassiana*. (**A−C**) Diagram for the deletion strategy of *frq1*, *frq2* and *frh* and their mutants identified through PCR (lanes 1−3) and southern blotting (lanes 4−6) analyses with paired primers and amplified probes (Table S1), respectively. Lanes 1 and 4: wild-type. Lanes 2 and 5: the deletion mutant. Lanes 3 and 6: the complemented mutant. Genomic DNAs were digested with *Pvu*I/*Pvu*I, *Eco*RV/*Eco*RI and *Pvu*I/*Pvu*I at the indicated sites for southern blotting of *frq1*, *frq2* and *frh*, respectively.

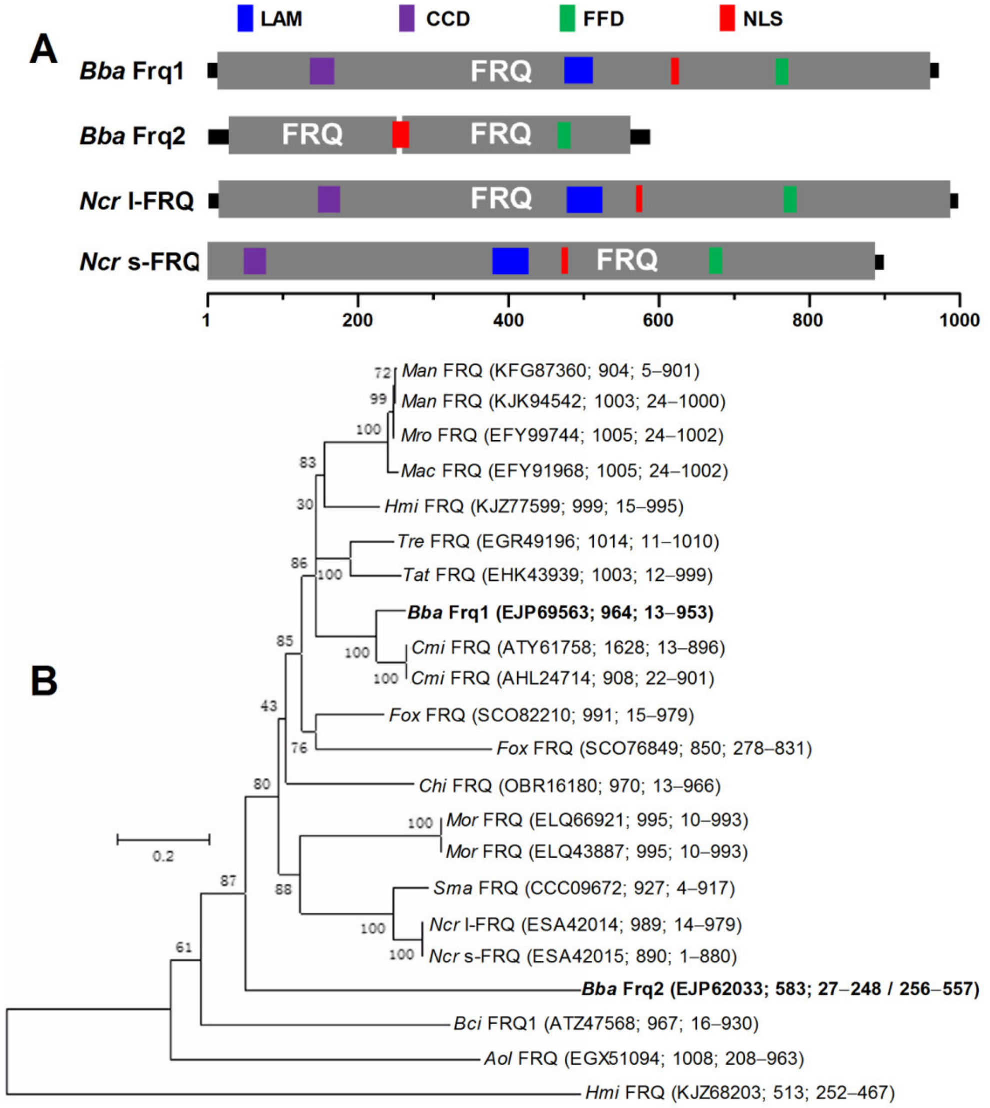

Figure S2. Bioinformatic analysis of two FRQ proteins (Frq1 and Frq2) in *Beauveria bassiana* (*Bba*). (**A**) Structural comparison of *Bba* Frq1 and Frq2 with l-FRQ and s-FRQ in *Neurospora crassa* OR74A (*Ncr*). (**B**) Phylogenetic relationships of *Bba* Frq1 and Frq2 with their homologs found in the genomic databases of other representative fungi (*Aol*, *Arthrobotrys oligospora* ATCC 24927; *Bci*, *Botrytis cinerea*; *Chi*, *Colletotrichum higginsianum* IMI 349063; *Cmi*: *Cordyceps militaris* CM581 / *C. militaris* CM141; *Fox*, *Fusarium oxysporum* V64-1; *Hmi*, *Hirsutella minnesotensis* 3608; *Mac*, *Metarhizium acridum* CQMa 102; *Man*, *Metarhizium anisopliae* / *M. anisopliae* BRIP 53284; *Mor*, *Magnaporthe oryzae* P131 / *M. oryzae* Y34; *Mro*, *Metarhizium robertsii* ARSEF 23; *Sma*, *Sordaria macrospora* k-hell; *Tat*, *Trichoderma atroviride* IMI 206040; *Tre*, *Trichoderma reesei* QM6a). Each FRQ protein is followed by the code of its NCBI accession, the number of its amino acids and the location of its FRQ domain in parentheses respectively. The bootstrap values of 1000 replications are given at nodes. Scale: branch length proportional to genetic distance assessed with the neighbor-joining method in MEGA7 software at http://www.megasoftware.net.

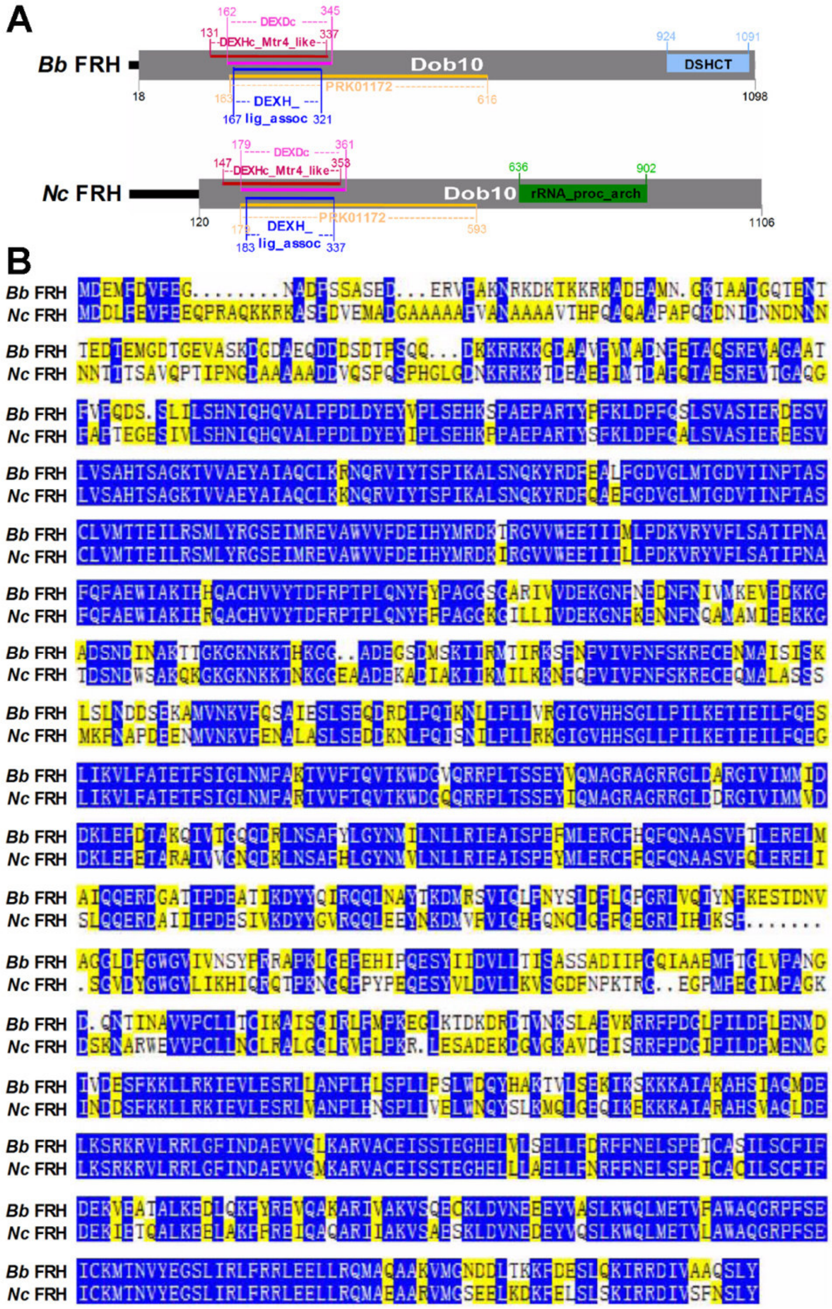

Figure S3. Comparison of FRH orthologs in *B. bassiana* (*Bb*) and *N. crassa* (*Nc*). (**A**) Main domains predicted from the sequences of *Bb* FRH and *Nc* FRH. (**B**) Distribution of identical (in blue) and similar (in yellow) amino acids in the sequences of *Bb* FRH and *Nc* FRH.

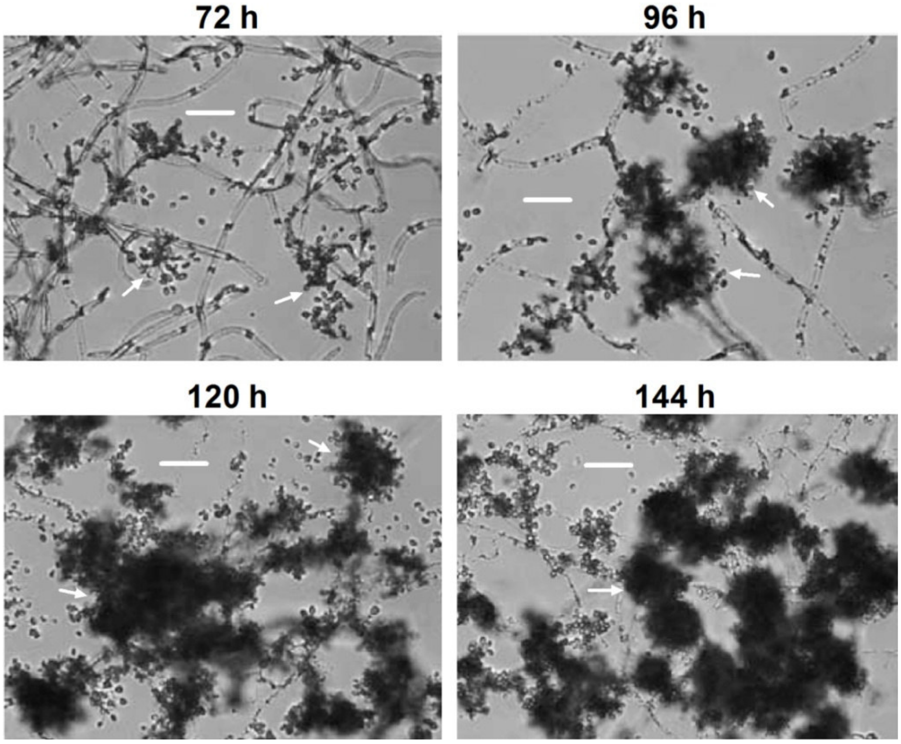

Figure S4. Microscopic images for conidiation status of wild-type *B. bassiana* strain during normal cultivation. The examined samples were taken from the SDAY cultures initiated by spreading 100 μl of a 10^7 conidia cm^{-2} suspension per plate (9 cm diameter) and incubated for 72−144 h at the optimal regime of 25°C in an L:D 12:12 cycle. The arrows indicate spore balls (conidia and zigzag rachises) formed on sampling occasions indicated. Scale bar: 20 μm.
